# The Wnt pathway protein Dvl1 targets somatostatin receptor 2 for lysosome-dependent degradation

**DOI:** 10.1016/j.jbc.2023.104645

**Published:** 2023-03-23

**Authors:** Heather S. Carr, Yan Zuo, Jeffrey A. Frost

**Affiliations:** Department of Integrative Biology and Pharmacology, University of Texas Health Science Center, Houston, Texas, USA

**Keywords:** Sstr2, G protein-coupled receptor (GPCR), Somatostatin, Dvl1, Wnt, lysosome, cell signaling, ubiquitin

## Abstract

The Somatostatin receptor 2 (Sstr2) is a heterotrimeric G protein-coupled receptor that is highly expressed in neuroendocrine tumors and is a common pharmacological target for intervention. Unfortunately, not all neuroendocrine tumors express Sstr2, and Sstr2 expression can be downregulated with prolonged agonist use. Sstr2 is rapidly internalized following agonist stimulation and, in the short term, is quantitatively recycled back to the plasma membrane. However, mechanisms controlling steady state expression of Sstr2 in the absence of agonist are less well described. Here, we show that Sstr2 interacts with the Wnt pathway protein Dvl1 in a ligand-independent manner to target Sstr2 for lysosomal degradation. Interaction of Sstr2 with Dvl1 does not affect receptor internalization, recycling, or signaling to adenylyl cyclase but does suppress agonist-stimulated ERK1/2 activation. Importantly, Dvl1-dependent degradation of Sstr2 can be stimulated by overexpression of Wnts and treatment of cells with Wnt pathway inhibitors can boost Sstr2 expression in neuroendocrine tumor cells. Taken together, this study identifies for the first time a mechanism that targets Sstr2 for lysosomal degradation that is independent of Sstr2 agonist and can be potentiated by Wnt ligand. Intervention in this signaling mechanism has the potential to elevate Sstr2 expression in neuroendocrine tumors and enhance Sstr2-directed therapies.

The Somatostatin receptor 2 (Sstr2) is a heterotrimeric G protein-coupled receptor that is highly expressed in neuroendocrine tissues, where it often functions to limit hormone secretion. High Sstr2 expression is also a defining feature in many neuroendocrine tumors, and as such Sstr2 agonists are often used to suppress the excess hormone secretion that is a common, debilitating feature in these tumors (1, 2, 3, 4, 5). In addition, a radionuclide-coupled Sstr2 agonist was recently approved for use as a tumor cell killing therapeutic in mid-gut neuroendocrine tumors, thus creating a clinical precedent for the conjugation of tumor cell killing analogs to Sstr2 agonists (6, 7, 8). Unfortunately, not all neuroendocrine tumors express significant levels of Sstr2 and prolonged agonist therapy can cause receptor downregulation. Thus, it is important to identify regulatory mechanisms controlling Sstr2 trafficking and expression.

Upon agonist addition, Sstr2 is rapidly internalized in a GRK- and β-arrestin-dependent manner. It is then trafficked to the Golgi, following which it is thought to be quantitatively recycled back to the plasma membrane (9, 10, 11, 12, 13, 14, 15, 16, 17, 18). Sstr2 trafficking may also be controlled by interaction with PDZ domain containing proteins, which bind to C-terminal PDZ domain-binding sites in many G protein-coupled receptors to regulate their trafficking and signaling potential (19, 20). The C-terminal PDZ domain-binding site of Sstr2 has been shown to bind to PDZ domain-containing proteins such as Shank1, Shank2, Magi1, and SYNJ2BP (21, 22, 23, 24). Moreover, we have demonstrated that interaction with SYNJ2BP enhances agonist-induced receptor internalization and signaling to the ERK/MAPK pathway (24).

In a screen for PDZ domain-containing proteins that may bind to Sstr2, we also identified Dishevelled 1 (Dvl1) as a potential-interacting protein (24). Dvl1 is a scaffolding protein that plays a key role in controlling β-catenin activation by the Wingless (Wnt) family of extracellular ligands (25, 26, 27, 28). It also targets the Frizzled (Fzd) family of Wnt receptors for lysosomal degradation, thereby restricting the extent of β-catenin activation (29, 30). There are 3 Dvl genes in the human genome, Dvl1, 2, and 3 and most cells express more than one Dvl protein. Importantly, binding of Dvl proteins to Fzd receptors recruits the ubiquitin E3 ligases RNF43 and ZNRF3, which then ubiquitylate Fzds to target them to the lysosome. Thus, this mechanism limits the number of Fzd receptors on the cell surface and negatively regulates Fzd-dependent signaling.

In the present work, we show that Dvl1 binds to Sstr2 in an agonist-independent manner, targeting Sstr2 for lysosomal degradation. Furthermore, we demonstrate that excess Wnt signaling promotes Sstr2 degradation and that small molecule inhibition of Dvl1 can enhance Sstr2 expression in neuroendocrine cells. These data indicate that Dvl1 limits Sstr2 expression in the absence of Sstr2 agonist and that excess Wnt signaling can exacerbate this process. These findings also suggest that limiting Dvl1 function may be a possible therapeutic approach to enhance Sstr2-directed therapies in patients with neuroendocrine tumors.

## Results

### Dvl1 interacts with Sstr2 to inhibit its expression

**Figure 1:**
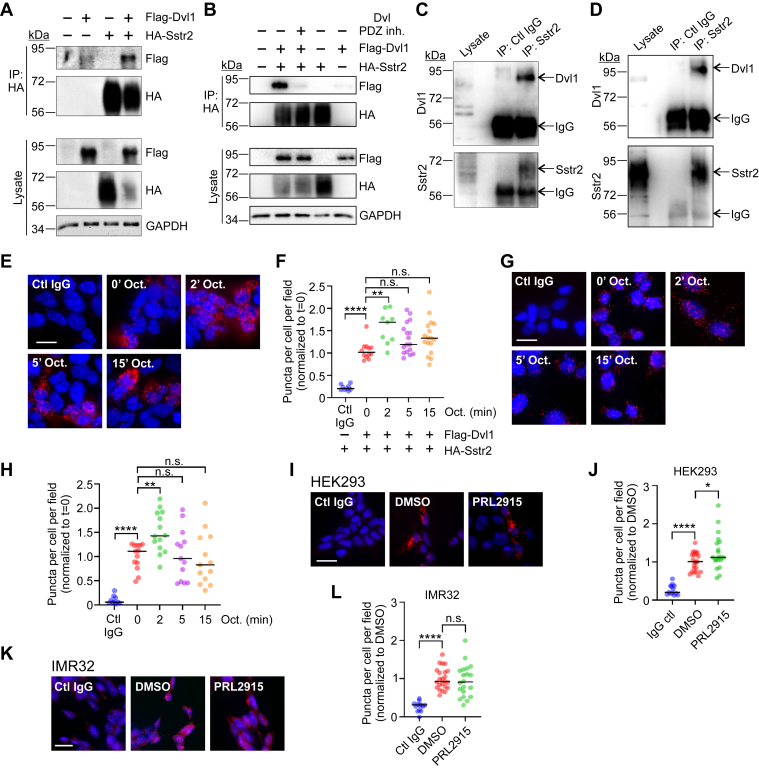
**Interaction of Dvl1 with Sstr2.***A*, HEK293 cells were transfected with HA-Sstr2 and Flag-Dvl1. HA-Sstr2 was immunoprecipitated and tested for Dvl1 coprecipitation by Western blotting. Shown is a representative experiment from three separate experiments. *B*, HEK293 cells were transfected with HA-Sstr2 and Flag-Dvl1 and then incubated with the pan-Dvl PDZ domain inhibitor NSC668036 overnight. HA-Sstr2 was then immunoprecipitated and tested for Dvl1 coprecipitation by Western blotting. Shown is a representative experiment from three separate experiments. *C*, endogenous Sstr2 was immunoprecipitated from IMR32 cells and tested for coprecipitation of endogenous Dvl1 by Western blotting. Shown is a representative experiment from three separate experiments. *D*, endogenous Sstr2 was immunoprecipitated from H69 cells and tested for coprecipitation of endogenous Dvl1 by Western blotting. Shown is a representative experiment from three separate experiments. *E*, HA-Sstr2 and Flag-Dvl1 transfected HEK293 cells were stimulated with octreotide (100 nM) for the times shown. Interaction was tested by proximity ligation assay (PLA). *Red dots* denote positive interactions. Nuclei are *blue*. Shown are representative micrographs. The scale bar represents 20 μm. *F*, quantification of PLA analysis of HA-Sstr2 and Flag-Dvl1 interaction in transfected HEK293 cells. Shown are the combined results from three separate experiments. Bars denote median values. *G*, interaction between endogenous Sstr2 and endogenous Dvl1 in IMR32 cells before and after octreotide stimulation, as assessed using PLA. Shown are representative micrographs. *Red* = positive PLA, *blue* = DNA. The scale bar represents 20 μm. *H*, quantification of PLA analysis in IMR32 cells. Shown are the combined results from three separate experiments. Bars denote median values. *I*, HA-Sstr2 and Flag-Dvl1 transfected HEK293 cells were tested for interaction using PLA, with or without prior incubation with the Sstr2 antagonist PRL2915 (100 nM, 30 min). Shown are representative micrographs. The scale bar represents 20 μm. *J*, quantification of PLA analysis in (*I*) after PRL2915 treatment. Shown are the combined results from three separate experiments. Bars denote median values. *K*, interaction of endogenous Sstr2 and Dvl1 in IMR32 cells was tested by PLA, with or without prior incubation with 100 nM PRL2915 for 30 min. Shown are representative micrographs. The scale bar represents 20 μm. *L*, quantification of PLA analyses in (*K*). Shown are the combined results of three separate experiments, and bars denote median values. Significance in (*F*), (*H*), (*J*), and (*L*) was assessed by one-way ANOVA with multiple comparisons, Dunnett’s post hoc test. ∗*p* < 0.05; ∗∗*p* < 0.01; ∗∗∗*p* < 0.001; ∗∗∗∗*p* < 0.0001. n.s. = not significant.

We have previously shown that a peptide corresponding to the C-terminal PDZ domain-binding site of Sstr2 interacted with the PDZ domain of Dvl1 (24). To determine whether full-length Sstr2 and Dvl1 interact, HEK293 cells were transfected with HA-epitope tagged Sstr2 and Flag-tagged Dvl1. HA-Sstr2 was then immunoprecipitated and tested for coprecipitation of Flag-Dvl1 by Western blotting. We observed that Dvl1 efficiently coimmunoprecipitated with Sstr2, indicating that the full-length proteins interact in cells (Fig. 1*A*). To test whether this required the PDZ domain of Dvl1, transfected cells were incubated with a pan-Dvl PDZ domain inhibitor NSC668036 (31) prior to lysis and immunoprecipitation of HA-Sstr2. We found that NSC668036 effectively blocked coprecipitation of Flag-Dvl1 (Fig. 1*B*). To determine whether the endogenous proteins interact, we tested for coimmunoprecipitation of Dvl1 with Sstr2 in the neuroendocrine cell lines IMR32 and H69. Importantly, we found that endogenous Dvl1 coimmunoprecipitated with endogenous Sstr2 in both of these cell lines (Fig. 1, *C* and *D*).

We then examined whether the interaction between Sstr2 and Dvl1 was influenced by Sstr2 agonist. We used proximity ligation assays (PLA) for this analysis, as a portion of agonist-stimulated Sstr2 is rapidly internalized into vesicles that are insoluble in buffers suitable for coimmunoprecipitation assays (24). HEK293 cells were transfected with HA-Sstr2 and Flag-Dvl1 and then stimulated for different times with the Sstr2 agonist octreotide. Cells were then fixed in paraformaldehyde (PFA) and processed for PLA. We observed that there was a basal level of interaction that was increased 2 min after agonist addition and largely returned to baseline after 5 min (Fig. 1, *E* and *F*). To ascertain whether this was also the case for the endogenous proteins, a similar experiment was performed in IMR32 cells. In these cells we observed a significant interaction of endogenous Dvl1 and Sstr2 in the absence of agonist that was also slightly increased 2 min after agonist addition and returned to baseline by 5 min (Fig. 1, *G* and *H*).To determine whether trace amounts of agonist potentially present in serum were promoting interaction between Sstr2 and Dvl1, transfected HEK293 cells and IMR32 cells were incubated with the Sstr2 antagonist PRL2915 prior to fixation. We found that incubation with PRL2915 caused a very small but statistically significant increase in interaction between HA-Sstr2 and Flag-Dvl1 in transfected HEK293 cells but was without effect for the endogenous proteins in IMR32 cells (Fig. 1, *I*–*L*). We conclude that Sstr2 and Dvl1 interact in the absence of receptor activation and that agonist stimulation causes a small, transient increase in this interaction.

### Dvl1 inhibits Sstr2 expression

**Figure 2:**
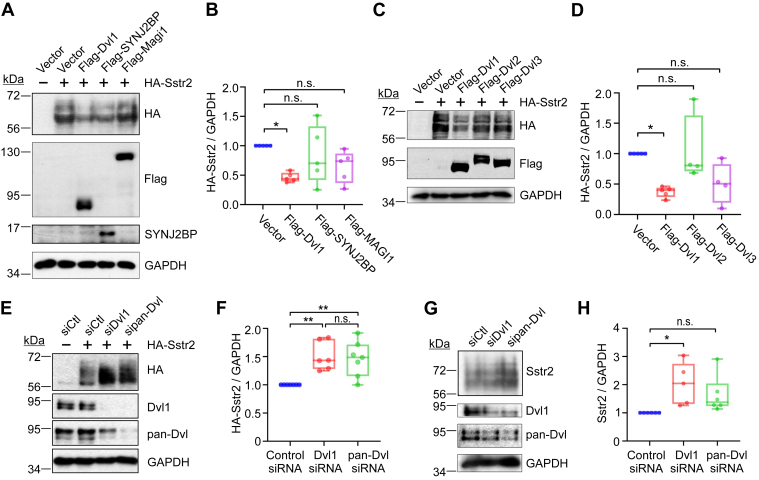
**Dvl1 inhibits Sstr2 expression.***A*, HEK293 cells were transfected with HA-Sstr2 and the Flag-tagged PDZ domain-containing proteins shown. Cells were lysed in an SDS- and urea-containing buffer (whole cell lysate) and HA-Sstr2 expression was assessed by Western blotting. Shown is a representative experiment from five separate experiments. *B*, quantification of HA-Sstr2 expression after cotransfection of PDZ domain-containing proteins. Bars denote median values. *C*, HEK293 cells were transfected with HA-Sstr2 and Flag-tagged Dvl1, Dvl2, or Dvl3. HA-Sstr2 expression in whole cells lysates was assessed by Western blotting. Shown is a representative experiment from four separate experiments. *D*, quantification of HA-Sstr2 expression after cotransfection of Flag-tagged Dvl1, 2, or 3. Each point represents the average number of puncta per cell in a field, with a minimum of three separate fields quantified per experiment. Bars denote median values. *E*, HEK293 cells were transfected with control, Dvl1, or pan-Dvl-specific siRNAs. One day later the cells were retransfected with HA-Sstr2. HA-Sstr2 expression was examined by Western blotting. Shown is a representative experiment from nine separate experiments. *F*, quantification of HA-Sstr2 expression after control, Dvl1, or pan-Dvl knockdown. Bars denote median values. *G*, IMR32 cells were transfected with control, Dvl1, or pan-Dvl siRNAs and tested for effects on endogenous Sstr2 expression. Shown is a representative experiment from six separate experiments. *H*, quantification of Sstr2 expression after Dvl1 or pan-Dvl knockdown. Each point represents the average number of puncta per cell in a field, with a minimum of three separate fields quantified per experiment. Bars denote median values. Significance in (*B*), (*D*), and (*H*) was assessed by one-way ANOVA with multiple comparisons, Dunnett’s post hoc test. Significance in (*F*) was assessed by unpaired Student’s *t* test. ∗*p* < 0.05; ∗∗∗*p* < 0.001. n.s. = not significant.

We observed that coexpression of Dvl1 with Sstr2 consistently reduced Sstr2 expression in the detergent soluble fraction (Fig. 1*A*). To determine whether this was due to the movement of Sstr2 to an insoluble fraction, HEK293 cells transfected with HA-Sstr2, minus or plus Dvl1. As controls, we also tested the effects of overexpressing the PDZ domain-containing proteins SYNJ2BP and Magi1, which we have shown to interact with the PDZ domain-binding site of Sstr2 (24). The cells were then lysed in an SDS- and urea-containing buffer to solubilize all cellular proteins and tested for HA-Sstr2 expression by Western blotting. We found that coexpression of Dvl1, but not SYNJ2BP or Magi1, significantly reduced Sstr2 expression, indicating that the reduced expression of Sstr2 was not due to the movement of the receptor to a detergent insoluble domain (Fig. 2, *A* and *B*).

There are three Dvl proteins in human cells, Dvl1-3. To determine whether any Dvl family member could inhibit Sstr2 expression, HEK293 cells were transfected with HA-Sstr2 and Flag-tagged Dvl1, 2 or 3, lysed in SDS/urea-containing buffer, and analyzed by Western blotting. We observed that only Dvl1 was capable of inhibiting Sstr2 expression. Overexpression of Dvl3 somewhat reduced Sstr2 expression but this did not reach statistical significance. Dvl2 overexpression was largely without effect (Fig. 2, *C* and *D*). These data agree with our observation that only the recombinant PDZ domain of Dvl1, but not Dvl2 or Dvl3, interacted with the C-terminal binding site of Sstr2 (24). To determine whether endogenous Dvl1 inhibited Sstr2 expression, HEK293 cells were transfected with control, Dvl1-specific, or pan-Dvl-targeting (targets a region common to all three Dvl genes) siRNAs. One day later the cells were then retransfected with HA-Sstr2, lysed in SDS-urea buffer, and analyzed by Western blotting. We found that Dvl1 or pan-Dvl knockdown significantly enhanced HA-Sstr2 expression (Fig. 2, *E* and *F*). These data indicate that endogenous Dvl1 limits transfected Sstr2 expression in HEK293 cells. Moreover, the pan-Dvl siRNA was not any more efficient at enhancing HA-Sstr2 expression, indicating that additional Dvl proteins do not participate in this regulation. To determine whether endogenous Dvl proteins inhibit the expression of endogenous Sstr2, IMR32 cells were transfected with Dvl1-specific or pan-Dvl siRNAs (32), lysed in SDS-urea buffer, and tested for Sstr2 expression by Western blotting. Similar to HEK293 cells, we observed that knockdown of Dvl1 alone was sufficient to enhance endogenous Sstr2 expression (Fig. 2, *G* and *H*).

### Dvl1 targets Sstr2 to the lysosome

**Figure 3:**
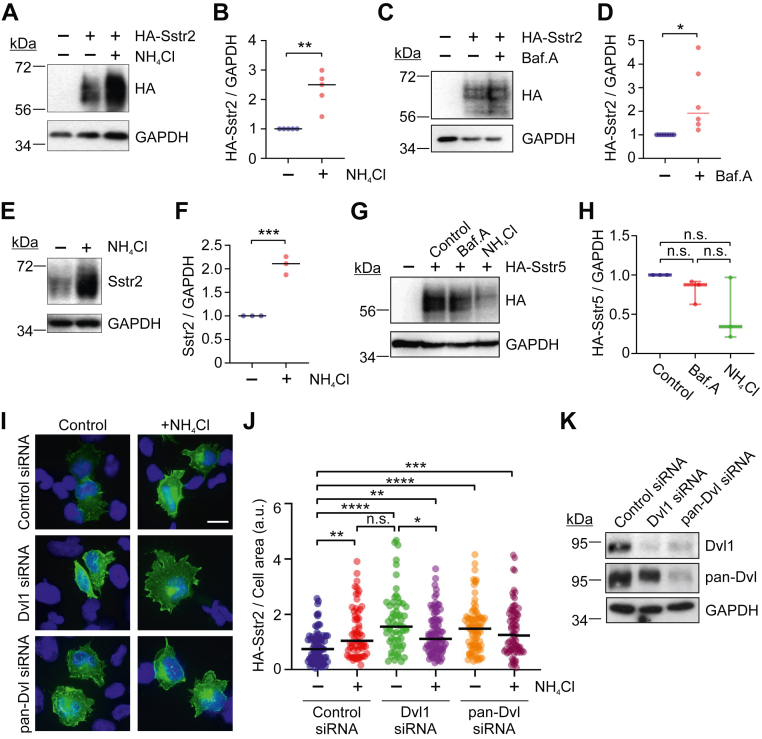
**Sstr2 is degraded by the lysosome in the absence of agonist.***A*, HEK293 cells were transfected with HA-Sstr2 and then treated for 2 h with the lysosome inhibitor NH_4_Cl. Whole cell lysates were tested for HA-Sstr2 expression by Western blotting. Shown is a representative experiment from four separate experiments. *B*, quantification of HA-Sstr2 expression after NH_4_Cl treatment. Bars are median values. *C*, HEK293 cells were transfected with HA-Sstr2 and treated for 2 h with bafilomycin A. Shown is a representative experiment from six separate experiments. *D*, quantification of HA-Sstr2 expression after bafilomycin treatment. Bars are median values. *E*, IMR32 cells were treated for 2 h with NH_4_Cl and assessed for endogenous Sstr2 expression by Western blotting. Shown is a representative experiment from three separate experiments. *F*, quantification of endogenous Sstr2 expression in IMR32 cells after NH_4_Cl treatment. Bars are median values. *G*, HEK293 cells were transfected with HA-Sstr5 and treated for 2 h with bafilomycin A or NH_4_Cl. Shown is a representative experiment from three separate experiments. *H*, quantification of HA-Sstr5 expression after bafilomycin or NH_4_Cl treatment. Bars are median values. *I*, HEK293 cells were transfected with control, Dvl1, or pan-Dvl-specific siRNAs, and then retransfected with HA-Sstr2. Prior to fixation, the cells were treated with NH_4_Cl for 2 h. Shown are representative micrographs, all taken with the same exposure time. *Green* = HA-Sstr2; *blue* = DNA. The scale bar represents 20 μm. *J*, quantification of HA-Sstr2 expression normalized to cell area. *Dots* represent values for individual cells. Results are combined from three separate experiments. Bars represent median values. *K*, Western blots of whole cell lysates were analyzed by western to validate Dvl1 or pan-Dvl silencing. Significance for (*B*), (*D*), and (*F*) was determined with unpaired student’s *t* test. Significance for (*H*) and (*J*) was determined by one-way ANOVA with multiple comparisons, Tukey’s post hoc test. ∗*p* < 0.05; ∗∗*p* < 0.01; ∗∗∗∗*p* < 0.0001; n.s. = not significant.

Dvl proteins are known to limit the expression of the Fzd family of Wnt receptors by targeting them to the lysosome for degradation (29, 30). Although Sstr2 is generally not recognized to traffic to lysosomes after agonist stimulation, it has not been well considered whether the receptor is subject to lysosomal degradation in the absence of Sstr2 ligand. To assess whether unstimulated Sstr2 is degraded in lysosomes, HEK293 cells were transfected with HA-Sstr2 and then treated with the lysosomal inhibitor ammonium chloride (NH_4_Cl). Cells were lysed in SDS-urea buffer and analyzed by Western blotting. We observed that treatment of cells with ammonium chloride for just 2 h significantly enhanced HA-Sstr2 expression (Fig. 3, *A* and *B*). To confirm that lysosomal inhibition was necessary to elevate Sstr2 expression, we treated Sstr2-transfected cells with the lysosome inhibitor bafilomycin A. We found that it was also effective at increasing HA-Sstr2 expression, indicating that the enhanced HA-Sstr2 expression observed with ammonium chloride treatment was the result of lysosome inhibition (Fig. 3, *C* and *D*). To determine whether lysosome inhibition boosted endogenous Sstr2 expression, we treated IMR32 cells with ammonium chloride for 2 h. Similar to transfected HA-Sstr2, we found that a 2-h treatment of these cells with ammonium chloride significantly elevated endogenous Sstr2 expression (Fig. 3, *E* and *F*). To confirm that this effect was specific for Sstr2, HEK293 cells were transfected with HA-Sstr5 and then treated with either ammonium chloride or bafilomycin A. Neither of these treatments increased HA-Sstr5 expression, indicating that Sstr2 is specifically degraded in the lysosome in unstimulated cells (Fig. 3, *G* and *H*).

We then assessed whether knockdown of Dvl1 was able to elicit similar effects as lysosomal inhibition at boosting HA-Sstr2 expression. HEK293 cells were transfected with control, Dvl1-specific, or pan-Dvl siRNAs. One day later the cells were transfected with HA-Sstr2, following which the cells were fixed and stained for HA-Sstr2. The intensity of HA-Sstr2 was assessed by epifluorescence microscopy and quantified. These experiments showed that knockdown of Dvl1 significantly increased HA-Sstr2 expression and that this effect was not further enhanced by lysosome inhibition (Fig. 3, *I*–*K*).

**Figure 4:**
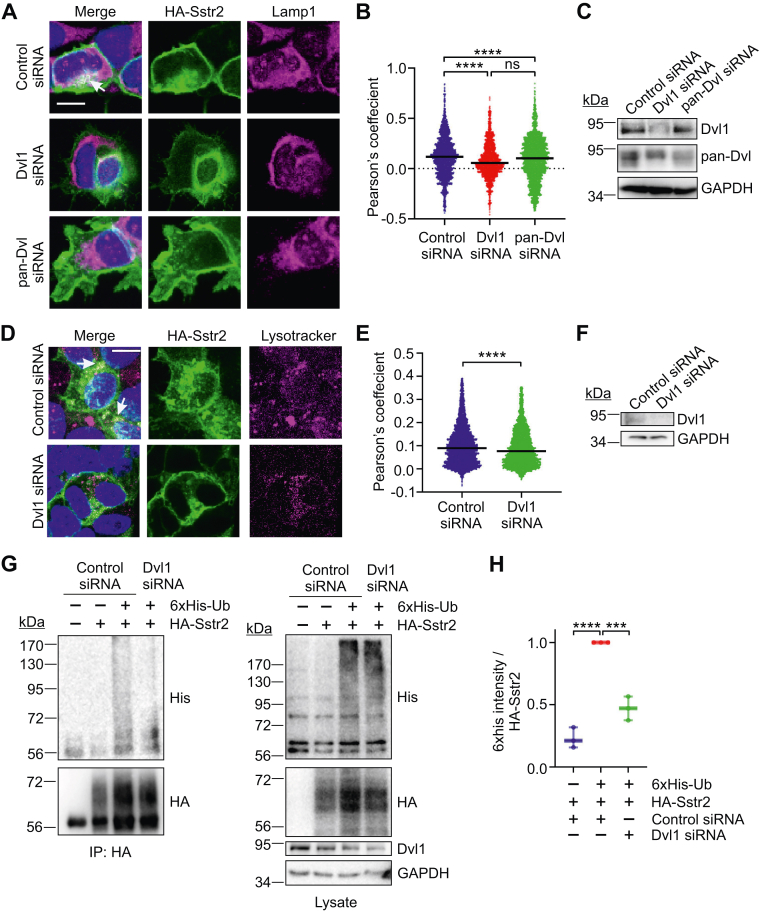
**Dvl1-dependent trafficking of Sstr2 to the lysosome.***A*, HEK293 cells were transfected with control, Dvl1, or pan-Dvl-specific siRNAs, after which they were transfected with HA-Sstr2. Cells were treated with NH_4_Cl for 2 h and then fixed and stained for HA-Sstr2 (*green*), Lamp1 (*magenta*), and DNA (*blue*). Images were acquired using confocal microscopy. Shown are representative z-plane images. The scale bar represents 10 μm. *B*, quantification of HA-Sstr2 and Lamp-1 colocalization using Pearson’s coefficient. Colocalization was calculated using the entire z-plane for each cell, with a minimum of 3 z-planes quantified per cell. A minimum of five cells per field were quantified, with a minimum of three fields assessed per experimental condition. Three biological replicates of each experimental condition were performed. *C*, representative Western blot of control, Dvl1, or pan-Dvl siRNA transfected HEK293 cells. *D*, HEK293 cells were transfected as in (*A*), treated for 2 h with 50 nM Lysotracker-Red, then fixed and stained for HA-Sstr2 (*green*) and DNA (*blue*). Images were acquired using confocal microscopy. Shown are representative z-plane images. The scale bar represents 10 μm. *E*, quantification of Lysotracker and HA-Sstr2 colocalization (Pearson’s coefficient) from three separate experiments. *F*, representative Western blot of control and Dvl1 siRNA transfected cells. *G*, HEK293 cells were transfected with control or Dvl1-specific siRNAs and then retransfected with HA-Sstr2 and 6xHis-Ub. Cells were lysed in radioimmunoprecipitation assay (buffer), immunoprecipitated for HA-Sstr2, and washed with 1 M NaCl and 0.5 M LiCl. Samples were tested for the 6xHis epitope by Western blotting and reblotted for HA-Sstr2. Shown is a representative experiment from three separate experiments. *H*, quantification of ubiquitylated HA-Sstr2 from three separate experiments. Ubiquitylated proteins greater than 72 kDa were quantified and normalized to HA-Sstr2 in the immunoprecipitates. Significance in (*B*) and (*H*) was determined by one-way ANOVA with multiple comparisons, Tukey’s post hoc test. Significance in (*F*) was determined with unpaired Student’s *t* test. ∗∗∗*p* < 0.001; ∗∗∗∗*p* < 0.0001.

To confirm that Sstr2 trafficked to the lysosome in a Dvl1-dependent manner, HEK293 cells were transfected with control, Dvl1-specific, or pan-Dvl siRNAs. One day later the cells were retransfected with HA-Sstr2 and then fixed and stained for HA-Sstr2 and the lysosomal protein Lamp1. Colocalization with Lamp1 was assessed by confocal microscopy. We found that Sstr2 colocalized with Lamp1 in the absence of ligand stimulation and that transfection of either Dvl1-specific or pan-Dvl siRNAs significantly reduced colocalization of Sstr2 with lysosomes (Fig. 4, *A*–*C*). Similar experiments in which lysosomes were labeled with Lysotracker recapitulated these results, confirming that Dvl1 targets Sstr2 to the lysosome (Fig. 4, *D*–*F*).

Sstr2 belongs to the seven transmembrane receptor superfamily of genes. Other members of this family that traffic to the lysosome are typically ubiquitylated prior to lysosome trafficking. To test whether this is the case for Sstr2, HEK293 cells were transfected with control or Dvl1 siRNAs. One day later the cells were transfected HA-Sstr2, plus or minus 6xHis tagged ubiquitin. Prior to lysis, the cells were incubated with ammonium chloride to inhibit the lysosome, after which they were lysed in radioimmunoprecipitation assay (buffer). The HA-Sstr2 was then immunoprecipitated, washed with 1 M NaCl and 0.5 M LiCl to remove nonspecific binding of ubiquitylated proteins, and tested for ubiquitylation by Western blotting. We found that knockdown of Dvl1 blocked Sstr2 ubiquitylation (Fig. 4, *G* and *H*).

**Figure 5:**
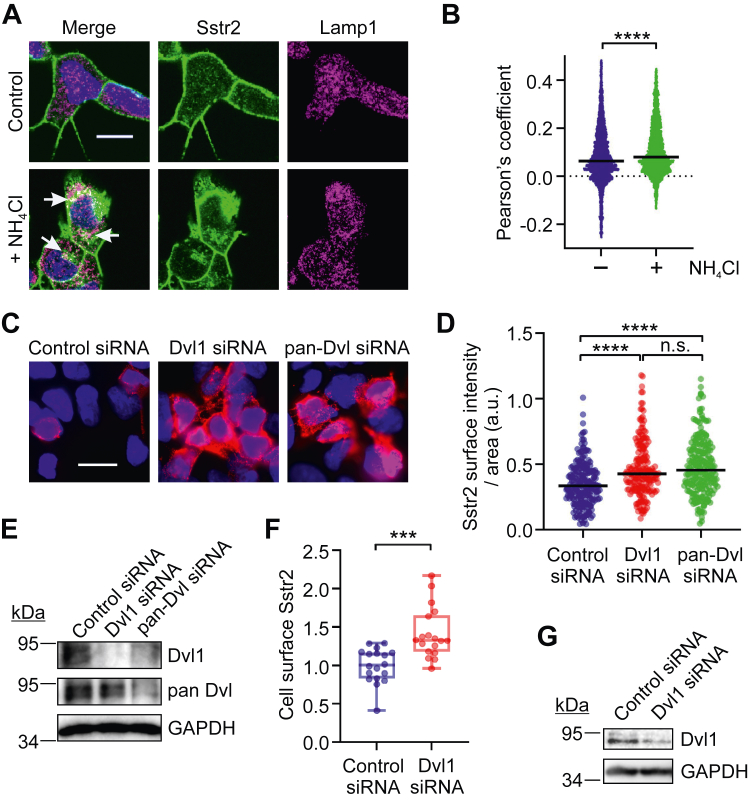
**Lysosomal targeting of endogenous Sstr2 by Dvl1.***A*, IMR32 cells were treated with vehicle or 20 mM NH_4_Cl for 2 h and then fixed and stained for Sstr2 (*green*), Lamp1 (*magenta*), and DNA (*blue*). Shown are representative z-plane images. The scale bar represents 10 μm. *B*, quantification of colocalization of Sstr2 and Lamp1 using Pearson’s coefficient, from three separate experiments. Bars are median values. *C*, IMR32 cells were transfected with the siRNAs shown and then fixed and stained for cell surface Sstr2 (*red*) and DNA (*blue*). Shown are representative micrographs. The scale bar represents 20 μm. *D*, quantification of Sstr2 surface expression divided by cell area in IMR32 cells. Each *dot* represents one cell. Results are combined from three separate experiments. *E*, representative Western blots for control, Dvl1, or pan-Dvl siRNA transfected IMR32 cells. *F*, quantification of cell surface Sstr2 in IMR32 cells by whole cell ELISA is shown. Three experiments with six replicates per condition are combined. *G*, representative Western blots for Dvl1 expression in siRNA transfected IMR32 cells used for cell surface ELISAs. Significance in (*B*) and (*F*) was determined with unpaired Student’s *t* test. Significance in (*D*) was determined with one-way ANOVA with multiple comparisons, Tukey’s post hoc test. ∗∗∗*p* < 0.001; ∗∗∗∗*p* < 0.0001; n.s. = not significant.

To test whether endogenous Sstr2 also trafficked to the lysosome in the absence of agonist, IMR32 cells were incubated with ammonium chloride and then fixed and stained for Sstr2 and Lamp1. These experiments showed that endogenous Sstr2 also accumulates in lysosomes following lysosome inhibition (Fig. 5, *A* and *B*). To test whether inhibition of lysosomal trafficking of Sstr2 resulted in an increase in cell surface Sstr2, IMR32 cells were transfected with control, Dvl1, or pan-Dvl targeting siRNAs, and then prebound to Sstr2 antibody to detect only surface receptor. The cells were then fixed and stained for Sstr2. We observed that knockdown of either endogenous Dvl1 or all Dvl isoforms significantly increased endogenous Sstr2 staining on the plasma membrane of IMR32 cells. Importantly, we observed that knockdown of Dvl1 was as effective as pan-Dvl knockdown, indicating that Dvl1 was mainly responsible for inhibiting cell surface expression of Sstr2 in these cells (Fig. 4, *C*–*E*). As an alternative way to measure cell surface expression of Sstr2, IMR32 cells were transfected with control or Dvl1-targeting siRNAs and then tested for Sstr2 expression on the cell surface by ELISA. These experiments confirmed that Dvl1 knockdown increased endogenous Sstr2 localization at the cell surface (Fig. 5, *F* and *G*). These data support the idea that Dvl1 is recruited to Sstr2 in an agonist-independent manner to promote Sstr2 trafficking to the lysosome.

### Dvl isoforms do not affect agonist-stimulated Sstr2 trafficking or signaling to Gi

**Figure 6:**
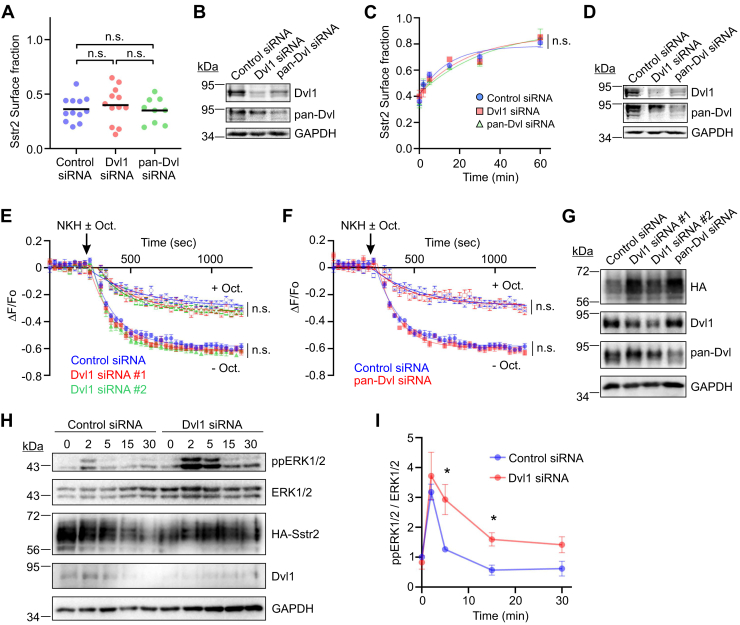
**Effects of Dvl1 knockdown on octreotide-stimulated Sstr2 internalization, recycling, and signaling.***A*, HEK293 cells were transfected with the siRNAs shown and then retransfected with HA-Sstr2. The cells were incubated with HA-epitope antibody and then treated with octreotide for 30 min. Surface fraction of HA-Sstr2 was determined by ELISA of nonpermeabilized cells. Shown are the results from four separate experiments, each done in triplicate. Significance was assessed by ANOVA with multiple comparisons, Tukey’s post hoc test. *B*, representative Western blot of HEK293 cells transfected with the siRNAs shown. *C*, HEK293 cells were transfected with the siRNAs shown and then retransfected with HA-Sstr2. Octreotide (100 nM) was added for 30 min, after which the Sstr2 antagonist PRL1392 (100 nM) was added for the times shown. Cells were fixed and surface Sstr2 was measured by ELISA. Shown are the results from three separate experiments combined, each performed in triplicate. *D*, representative Western blot of siRNA transfected cells. *E* and *F*, HEK293 cells stably expressing HA-Sstr2 were transfected with the siRNAs shown and then infected with a BacMam expressing a cADDis cAMP reporter. Cells were treated with the forskolin analog NHK477 (10 μM), minus or plus octreotide (100 nM), and reporter fluorescence was measured in real time. Shown are the average of three separate experiments. *G*, representative Western blot of HEK293 cells transfected with the siRNAs shown. *H*, HEK293 stably expressing HA-Sstr2 were transfected with control or Dvl1-specific siRNAs and then stimulated with the agonist octreotide (100 nM) for the times shown. ERK1/2 activation was assessed by Western blot analysis. Shown is a representative experiment from three independent experiments. *I*, quantification of ERK1/2 activation in control or Dvl1 knockdown cells. Shown is the average of three independent experiments. Errors are SEM. Significance in (*C*), (*E*), and (*F*) was determined by one-way ANOVA. n.s. = not significant. Significance in (*I*) was determined by Student’s *t* test. ∗*p* < 0.05.

Sstr2 is rapidly internalized after agonist stimulation and follows a number of intracellular trafficking routes before being recycled back to the plasma membrane. To determine whether Dvl proteins affect receptor internalization, HEK293 cells were transfected with control, Dvl1, or pan-Dvl siRNAs, and then retransfected with HA-Sstr2. The next day the cells were stimulated with the Sstr2 agonist octreotide and Sstr2 internalization was measured. In these experiments, we did not observe a significant effect of Dvl isoform knockdown on maximal HA-Sstr2 internalization (Fig. 6, *A* and *B*). In similar experiments, we were unable to discern an effect on HA-Sstr2 recycling back to the plasma membrane (Fig. 6, *C* and *D*). These data indicate that Dvl isoforms do not affect agonist-stimulated Sstr2 internalization or recycling, consistent with data indicating that Dvl1 targets Sstr2 to the lysosome in the absence of agonist.

Sstr2 is a Gi-coupled receptor that inhibits cAMP production, which is critical to its ability to suppress excess hormone secretion in neuroendocrine tumors. To examine whether Dvl isoforms affect the ability of Sstr2 to suppress cAMP production, HEK293 cells stably expressing HA-Sstr2 were transfected with control, Dvl1, or pan-Dvl siRNAs. The cells were then infected with a BacMam virus expressing the cADDis cAMP sensor and stimulated with the adenylyl cyclase agonist NKH477, minus or plus the Sstr2 agonist octreotide. As expected, octreotide stimulation significantly inhibited NKH477-stimulated cAMP production. Notably, knockdown of Dvl isoforms did not affect the ability of Sstr2 to inhibit cAMP production (Fig. 6, *E*–*G*).

Sstr2 has a well-documented ability to stimulate ERK1/2 activation (24, 33). To determine whether Dvl1 affected signaling to ERKs, cells expressing HA-Sstr2 were transfected with control or Dvl1-targeting siRNAs and then stimulated with octreotide for different times. ERK1/2 activation was assessed by Western blotting. Surprisingly, we found that Dvl1 knockdown significantly enhanced the ability of Sstr2 to stimulate ERK1/2 activation (Fig. 6, *H* and *I*). Since Dvl1 does not appear to affect agonist-dependent Sstr2 internalization, trafficking, or signaling to G_i_, this suggests that cell surface expression of Sstr2 may be limiting for ERK1/2 activation, and Dvl1 that knockdown relieves this constraint.

### Wnt signaling inhibits Sstr2 expression

**Figure 7:**
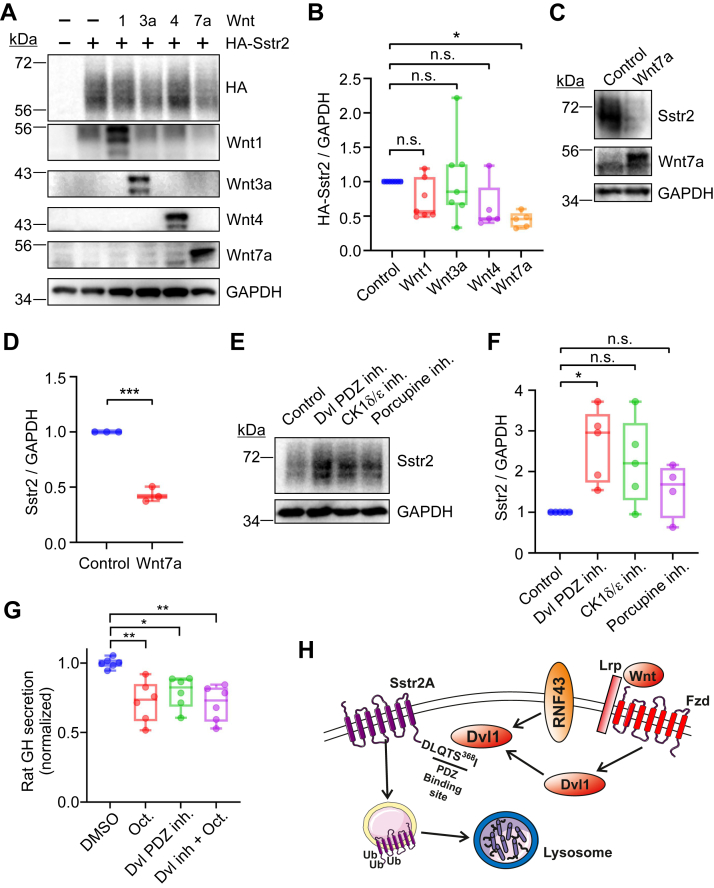
**Wnt signaling inhibits Sstr2 expression.***A*, HEK293 cells were transfected with HA-Sstr2 plus the Wnts shown. Whole cells lysates were examined for HA-Sstr2 expression by Western blotting. A representative experiment from five separate experiments is shown. *B*, quantification of HA-Sstr2 expression in Wnt transfected cells. Bars are median values. *C*, Western blot for endogenous Sstr2 expression in IMR32 cells stably expressing empty vector (control) or Wnt7a. Shown are representative Western blots. *D*, quantification of endogenous Sstr2 expression in control or Wnt7a expressing IMR32 cells. *E*, effects of overnight treatment with a pan-Dvl PDZ domain inhibitor (NSC668036, 10 μM), a CK1δ/ε inhibitor (PF670462, 10 μM), or a porcupine inhibitor (LGK974, 10 μM) on endogenous Sstr2 expression in IMR32 cells. *F*, quantification of Sstr2 expression in IMR32 cells after treatment with Wnt pathway inhibitors. Bars are the medians from 4 to 5 separate experiments. *G*, growth hormone (GH) secretion from GH3 cells was assessed following Dvl PDZ inhibitor treatment (10 μM, 4 h), in the presence and absence of octreotide (100 nM, 15 min). Shown is the average of three replicates performed in duplicate. *H*, model for regulation of Sstr2 trafficking to the lysosome by Dvl1. In (*B*) and (*F*), significance was assessed by ANOVA with multiple comparisons, Dunnett’s post hoc test. For (*G*), significance was assessed by ANOVA with Tukey’s multiple comparisons test. Significance in (*D*) was assessed by unpaired student’s *t* test. ∗*p* < 0.05; ∗∗*p* < 0.01; ∗∗∗*p* < 0.001; n.s. = not significant.

Our data indicates that Dvl1 promotes lysosomal degradation of Sstr2 in the absence of Sstr2 agonist stimulation. Dvl1 is normally activated by Wnt ligands to mediate intracellular signaling as well as Fzd downregulation (25, 26, 27, 28, 29, 30). Accordingly, we examined whether overexpression of Wnt ligands affected Sstr2 expression. There are 19 Wnt ligands that bind to 10 Fzd receptors with varying affinities (25). Wnts are generally grouped into canonical and noncanonical categories, based on whether they stimulate β-catenin activation. Although nearly all Wnt ligands stimulate Dvl isoform activation, they can differentially regulate the interaction of Dvl isoforms with accessory proteins to dictate which intracellular signaling pathways are activated (25, 26, 27). To begin to assess whether particular Wnts regulate Sstr2 expression, HEK293 cells were transfected with HA-Sstr2 and Wnt1, Wnt3a, Wnt4, and Wnt7a. Wnt1, Wnt3A, and Wnt7a are canonical ligands that potently stimulate β-catenin activation. They bind to an overlapping spectrum of Fzd receptors that together account for the majority of Fzd receptors except Fzd3, 6, and 9 (34). Wnt4 is a noncanonical ligand that acts through Fzd receptors to stimulate the Calcium/CAMK and planar cell polarity pathways and to a lesser extent the β-catenin pathway (35). We observed that overexpression of each of the four Wnts tended to inhibit Sstr2 expression but only Wnt7a overexpression caused a statistically significant effect (Fig. 7, *A* and *B*). These data indicate that Wnt stimulation of Fzd receptors can inhibit Sstr2 expression and that there is significant specificity as to which ligands are most effective.

To assess whether Wnts are capable of stimulating endogenous Sstr2 downregulation in neuroendocrine tumor cells, we stably overexpressed Wnt7a in IMR32 cells and then assessed endogenous Sstr2 expression by Western blotting. We observed a strong downregulation of Sstr2 expression in the Wnt7a expressing cells (Fig. 7, *C* and *D*), indicating that Wnt signaling is also capable of downregulating endogenous Sstr2 expression in neuroendocrine tumor cells.

The level of Sstr2 expression in neuroendocrine tumors is a limiting factor in the use of Sstr2 agonists or theranostics in neuroendocrine tumor patients (3, 5, 36). We therefore tested whether Sstr2 expression in neuroendocrine tumor cells could be enhanced by treatment with small molecule inhibitors of Wnt pathway signaling. IMR32 cells were treated overnight with the pan-Dvl PDZ domain inhibitor NSC668036 (31), the CK1δ/ε inhibitor PF670462 (37), or the porcupine inhibitor LGK974 (38). We used a CK1δ/ε inhibitor, as CK1ε phosphorylates Dvl isoforms to promote their activation and also phosphorylates the Fzd coreceptors Lrp5/6 to enable signaling to β-catenin (39, 40, 41, 42, 43, 44, 45). LGK974 inhibits the porcupine acyltransferase, which conjugates an essential acyl group to all Wnt ligands, thereby enabling their production (38, 46). Consequently, this inhibitor should block endogenous Wnt ligand synthesis. We observed that both the pan-Dvl PDZ domain inhibitor and to a lesser extent the CK1δ/ε inhibitor significantly elevated endogenous Sstr2 expression in IMR32 cells. Surprisingly, the porcupine inhibitor was without effect (Fig. 7, *E* and *F*). However, the porcupine inhibitor did block Wnt3a-stimuated β-catenin transcriptional reporter activity, indicating that the drug was functional (not shown).

Sstr2 agonists are used to suppress excess growth hormone (GH) secretion in functional pituitary tumors (1, 2, 3, 4, 5). To assess whether Wnt pathway inhibitors are able to modulate Sstr2-dependent suppression of GH secretion, GH3 rat pituitary tumor cells were treated for 4 h with the pan-Dvl PDZ domain inhibitor and then stimulated for 15 min with octreotide. GH secretion into the media was assessed by ELISA. We observed that treatment with the Dvl PDZ domain inhibitor caused a significant reduction in GH secretion that was nearly as great as that stimulated by octreotide. However, combining the Dvl PDZ domain inhibitor with octreotide stimulation did not further suppress GH secretion (Fig. 7*G*). Since suppression of GH secretion by Sstr2 is thought to be mainly regulated by its ability to suppress cAMP production and we did not observe an effect of Dvl1 on octreotide stimulated Gi activation (Fig. 6, *E*–*G*), these data are consistent with the notion that Dvl proteins do not affect agonist-dependent signaling to Gi. However, these results do suggest that under conditions where agonist is limiting, Dvl inhibition may be useful to suppress hormone secretion. These data also suggest that treatment of neuroendocrine tumors with pan-Dvl PDZ domain or CK1δ/ε inhibitors might be viable options to enhance Sstr2 expression and potentiate Sstr2-directed cytotoxic therapies.

## Discussion

In the present work we have shown that Dvl1 targets Sstr2 for lysosomal degradation in the absence of Sstr2 agonist stimulation and that interfering with this process can significantly elevate Sstr2 expression (Fig. 7*H*). These findings have important implications for the treatment of neuroendocrine tumors, as Sstr2 agonists are first-line therapies used to suppress excess hormone secretion by a wide variety of neuroendocrine tumors. In addition, Sstr2 agonist-coupled PET imaging agents and high-energy radionuclides are commonly used as diagnostic and theranostic agents in neuroendocrine tumor patients. The primary limitation of all of these approaches is the requirement for Sstr2 expression on the tumor cell surface, as often Sstr2 expression is either initially lacking or becomes downregulated with agonist-directed therapy. As a result, approaches to promote cell surface expression of Sstr2 in neuroendocrine tumor cells would be impactful.

Our data indicate that lysosomal trafficking of Sstr2 does not occur after Sstr2 agonist addition, supporting the idea that Dvl1-dependent lysosomal trafficking represents a heterologous form of receptor regulation. This agrees with a large body of evidence indicating that Sstr2 is fully recycled back to the plasma membrane after agonist-induced internalization (9, 10, 11, 12, 13, 14, 15, 16, 17, 18). However, it is important to note that Sstr2 has been found to colocalize with lysosomes in human neuroendocrine tumors treated with octreotide (47). Moreover, it may be that Sstr2 is not always destined to be completely recycled back to the plasma membrane after agonist addition. For example, in the mouse pituitary tumor cell line AtT20, it was recently shown that extended treatment of cells with Sstr2 agonists leads to receptor degradation *via* the lysosome (48). Thus, although not common, agonist-stimulated trafficking of Sstr2 to the lysosome occurs in some cell types and it may be that this process involves Dvl1.

We showed that Dvl1 stimulates Sstr2 ubiquitylation, similar to what occurs to Fzd receptors following Wnt stimulation. In this case, Dvl proteins promote recruitment of the ubiquitin E3 ligases RNF43 and ZNRF3, which are plasma membrane integrated proteins. This process can be opposed by the Rspondin (RSPO) family of extracellular ligands, which bind simultaneously to the extracellular domains of RNF43/ZNRF3 and Lgr4, 5 or 6 to prevent the association of RNF43/ZNRF3 with Fzd receptors (49, 50). Given that Dvl1 also targets Sstr2 for ubiquitin-mediated trafficking to lysosomes, we hypothesize that a similar mechanism may operate here. If true, this suggests that neuroendocrine tumors that produce one or more RSPOs and Lgr4/5/6 would not downregulate Sstr2 expression, even if they are expressing significant levels of Wnt ligands.

Dvl family proteins have not been shown to regulate seven transmembrane spanning receptors other than the Fzd family of receptors. Thus, our finding that Dvl1 binds to Sstr2 and targets it for lysosomal degradation is unexpected and novel. The interaction between Sstr2 and Dvl1 is independent of Sstr2 agonist (Fig. 1) and Dvl knockdown did not affect agonist-driven receptor internalization or recycling (Fig. 6). Similarly, Dvl knockdown did not affect Sstr2-dependent inhibition of adenylyl cyclase, which is the main signaling pathway downstream of this receptor (Fig. 6). However, Dvl1 knockdown did potentiate agonist stimulated ERK1/2 activation (Fig. 6). This may indicate that receptor levels are limiting for ERK1/2 activation but not for signaling to G_i_. This sort of effect may reflect the highly efficient coupling of Sstr2 to Gi as compared to its ability to signal to ERK1/2. However, regardless of the underlying reasons, it seems clear that Dvl1 can suppress Sstr2 signaling in a pathway-specific manner. This finding is reminiscent of the reported role of Dvl2 in regulating survival signaling by the PAR-1 receptor after adenomatous polyposis coli stimulation (51). Since Sstr2 signals to other proteins such as GIRKs and SHP-1 (52, 53), it would be interesting to know the full spectrum of Sstr2-dependent signaling pathways modulated by Dvl1.

Wnts are well known to drive tumorigenesis and there is some evidence for elevated Wnt pathway signaling in neuroendocrine tumors. For example, expression of the Wnt decoy receptors sFRP2, sFRP4, and Wif1 are downregulated in pituitary tumors by epigenetic mechanisms (54, 55, 56). Since these proteins suppress Wnt signaling, it would be expected that their reduced expression would allow for a greater flux through the Wnt pathway. Alternatively, Wnt4 has been reported to be overexpressed in pituitary tumors (57). There are reports of elevated β-catenin activity in gastrointestinal tract neuroendocrine tumor cells, suggesting that Wnt signaling is also upregulated in these cells (58, 59, 60, 61). Wnt signaling has also been reported to be elevated by various mechanisms in neuroendocrine prostate tumors (62, 63, 64, 65). As a consequence, the mechanism we have identified here may be frequently operating in human neuroendocrine tumors to regulate Sstr2 expression.

Given the well-documented role of Wnts in cancer, there have been considerable efforts to develop small molecule inhibitors that block Wnt signaling (25, 26, 66). Our data suggest that some of these approaches may be useful to prevent Dvl1-dependent Sstr2 degradation. In particular, the pan-Dvl PDZ domain inhibitor NSC668036 was most effective at boosting Sstr2 expression (Fig. 7, *E* and *F*). This agent inhibits Wnt3a-dependent signaling in cells and in *Xenopus* embryos (31). It has also been reported to be active in rodents to block paclitaxel- and diabetes-induced neuropathy and to attenuate bleomycin-induced pulmonary fibrosis (67, 68, 69). However, this inhibitor has not been tested in neuroendocrine tumors or in human patients. In the future, it will be interesting to test whether this inhibitor can boost Sstr2 expression in neuroendocrine tumor models.

## Experimental procedures

### Materials

Anti-Flag mouse monoclonal M2 (#F1804, 1:1000 for Western blot, 1:250 for immunocytochemistry) and anti-Dvl1 rabbit polyclonal antibody (#D3570) were purchased from Millipore-Sigma. Anti-hemagglutinin (HA) epitope rabbit polyclonal (#3724S), anti-phospho (T202/Y204) ERK1/2 (#4370), anti-ERK1/2 (#9102), and anti-LAMP1 rabbit polyclonal (#3242) antibodies were from Cell Signaling Technology. Anti-HA epitope mouse monoclonal (Covance Research Products Inc Cat# MMS-101R-500; 1:10,000 for Western blot, 1:10,000 for cell surface ELISA (https://scicrunch.org/resources/Any/search?q=AB_10063630&l=AB_10063630)) was purchased from Biolegend. Mouse monoclonal anti-GAPDH was from ProteinTech (60004-1-Ig) (https://scicrunch.org/resources/Any/search?q=60004-1-Ig&l=60004-1-Ig). Anti-Sstr2 rabbit antibody was from Thermo Fisher Scientific (PA3-109) (https://scicrunch.org/resources/Any/search?q=PA3-109&l=PA3-109), as was control rabbit IgG (10500C) (https://scicrunch.org/resources/Any/search?q=AB_2532981&l=AB_2532981). Anti-6xHis tag was from Thermo Fisher Scientific (14-6657-82). Anti-Sstr2 mouse monoclonal antibody (#MAB4224) was from R&D Systems. Mouse monoclonal anti-pan-Dvl antibody (sc-166303) was from Santa Cruz Biotechnology. AlexaFluor secondary fluorescent antibodies were purchased from Thermo Fisher Scientific (A11004 and A11008). Octreotide and PRL2915 were purchased from Bachem. Jetprime and INTERFERin transfection reagents were purchased from Polyplus Transfection. The Dvl PDZ domain inhibitor NSC668036 and casein kinase 1δ/ε inhibitor PF670462 were obtained from Tocris and porcupine o-acyl transferase inhibitor LGK974 was purchased from MedChemExpress.

The triple HA-epitope tagged WT rat Sstr2 plasmid has been described (18, 70). The triple HA-epitope tagged human Sstr5 was from cdna.org. Flag-tagged human Dvl1 was constructed by PCR amplification of the cDNA purchased from DNASU (clone ID: HCD00663358) and ligated into the EcoRI and XhoI sites of the pcDNA3.1-Flag plasmid. Flag-Dvl2 was constructed by PCR amplification of the coding sequence of human Dvl2 (OriGene clone SC128046) to insert restriction sites and a stop codon, followed by digestion and ligation into the EcoRI and XhoI sites of pcDNA3.1-Flag. The plasmids expressing Flag-tagged Dvl3 (#16758) and Flag-tagged MAGI1 (#10714) were obtained from Addgene. Flag-tagged human SYNJ2BP was as described (24). pCMV-his-Ubiquitin plasmid was a kind gift from Jianping Jin.

### Cell culture and transfections

HEK293 cells (Cat#R70507, Thermo Fisher Scientific) were maintained in Dulbecco’s modified Eagle’s medium (DMEM, Lonza) with 10% fetal bovine serum at 37 °C in 5% CO_2_. IMR32 cells were maintained in DMEM with 10% fetal bovine serum at 37 °C in 5% CO_2_. H69 cells were maintained in RPMI with 10% fetal bovine serum at 37 °C in 5% CO_2_. GH3 cells were maintained in DMEM-F12 with 2.5% fetal bovine serum and 12.5% horse serum at 37 °C in 5% CO_2_. The generation of stably-transfected HA_3_-Sstr2 HEK293 cells has been described (70). Stably transfected Wnt7a-IMR32 cells were generated by transfection with pcDNA3-Wnt7a followed by selection with G418 at 1 mg/ml.

Transient transfections were performed using JetPrime. siRNA transfections were performed *via* reverse transfection using INTERFERin 48 to 72 h before experiments. Dvl1 or pan-Dvl silencing was performed using the siRNA duplexes SASI_Hs01_00142403 (5′GTCGGAGTAGGGATCTAA[dT][dT]-3′), SASI_Hs02_00337577 (5′-GCGACATGTTGCTGCAGGT[dT][dT]-3′) or 5′-AAGUCAACAAGAUCACCUUCU[dT][dT]-3′ (pan-Dvl) (32). The universal negative control siRNA (SIC-001, Millipore Sigma) was used to control for nonspecific effects of siRNA transfection.

### Immunoprecipitation and Western blotting

HEK293 cells were transfected with the relevant plasmids for 24 h, then rinsed and refed serum-free medium and treated with 100 nM octreotide at 37 °C for the times indicated. Cells were then placed on ice, washed with cold PBS, and scraped into cold PBS supplemented with 1 mM PMSF, and 100 nM okadaic acid. Cell pellets were solubilized using lysis buffer (50 mM Hepes pH 7.4, 150 mM NaCl, 5 mM EDTA, 3 mM EGTA, 10 mM sodium pyrophosphate, 10 mM NaF, 1 mM PMSF, 10 μg/ml leupeptin, 10 μg/ml pepstatin, 10 μg/ml aprotinin, 100 nM okadaic acid), with 2 mg/ml dodecyl β-maltoside, and 0.2% Triton X-100, for 30 min at 4 °C, and centrifuged (16,000*g*, 10 min). Anti-HA agarose (80 μl of a 50% slurry) was added to the lysates and incubated at 4 °C for 2 h with gentle rocking. Pellets were washed three times with lysis buffer, eluted in urea sample buffer (125 mM Tris–HCl pH 6.8, 4% SDS, 20% glycerol, 6 M urea), and resolved by 10% SDS-PAGE. Coimmunoprecipitations of endogenous proteins from H69 and IMR32 cells were performed as above with the following modifications: cells were harvested with lysis buffer containing 0.5% C_12_E_8_ and allowed to lyse for 30 min on ice before centrifugation, followed by clarification with 1 μg of nonspecific rabbit IgG and Protein A-Sepharose for 30 min at 4 °C with gentle rocking. Clarified lysates were probed with 1.5 μg of rabbit anti-Sstr2 or rabbit IgG for 2 h at 4 °C in the presence of Protein A-Sepharose before being washed three times with lysis buffer supplemented with NaCl to 175 mM. Samples were eluted with SDS-urea sample buffer and resolved by SDS-PAGE. Proteins were transferred to polyvinylidene difluoride membrane and blocked in 5% nonfat dry milk or bovine serum albumin (BSA) in Tris-buffered saline Tween 20 (20 mM Tris–HCl pH 7.6, 150 mM NaCl, 0.1% Tween20) as appropriate before incubation with the indicated primary at 4 °C overnight. Membranes were washed three times with Tris-buffered saline Tween 20 before incubation with a 1:20,000 dilution of horse radish peroxidase (HRP)-linked secondary antibody for 30 min at room temperature (RT). Blots were washed three times with Tris-buffered saline Tween 20 and rinsed with PBS before development with Pierce ECL reagent. For detection, either X-ray film or an Azure C280 Chemiluminescent Western Blot Imager was utilized. For the latter, images were saved as TIFFs; all images were analyzed in ImageJ (https://imagej.nih.gov/ij/index.html).

For ubiquitylation experiments, HEK293 cells were reverse transfected with control or Dvl1-specific siRNA and allowed to recover overnight, then transfected with HA-Sstr2 plasmid in the presence or absence of pCMV-his-Ub. On the day of the experiment, cells were treated with 20 mM NH_4_Cl for 2 h before harvest in radioimmunoprecipitation assay (buffer) supplemented with 2 mM *N*-ethylmaleimide, 2 mM PMSF, and 10 μM MG132. HA-Sstr2 was immunoprecipitated with mouse anti-HA (2 μg) and Protein A-Sepharose beads for 2 h at 4 °C before beads were pelleted and washed twice with 1 ml of 50 mM Tris–HCl pH 8.0, 1 M NaCl, 0.5% Triton X-100, 2 mM PMSF, and twice more with 1 ml of 50 mM Tris–HCl pH 8.0, 500 mM LiCl, 0.5% Triton X-100, 2 mM PMSF. Beads were resuspended in 40 μl SDS-urea sample buffer and frozen at −80 °C until Western analysis was performed.

### Proximity ligation assay

Interactions between Sstr2 and Dvl1 were analyzed by DuoLink PLA (Millipore Sigma). HEK293 or IMR32 cells were plated on coverslips coated with poly l-ornithine 1 day prior to transfection. HEK293 cells were transiently transfected with empty vector, HA-tagged Sstr2, and Flag-Dvl1 as indicated, 1 day prior to experiment. The day of the experiment, the coverslips were washed with DMEM plus 5 mg/ml lactalbumin hydrolysate (LH) and 20 mM Hepes (pH 7.4) (DMEM/LH/Hepes) and allowed to re-equilibrate at 37 °C, 5% CO_2_ for 15 to 20 min before being treated with 100 nM octreotide for the indicated times or 100 nM PRL2915, an Sstr2-specific antagonist, for 30 min. Coverslips were washed with cold PBS and fixed with 3% PFA at RT for 10 min. Cells were permeabilized with 0.3% Triton X-100 plus 1% BSA in PBS for 15 min at RT. PLA was performed according to the manufacturer’s instructions, with the following modifications: for better visualization of nuclei and cell edges, a 10 min incubation at RT with 1 μg/ml 4′-6-diamidino-2-phenylindole (DAPI) in the presence or absence of phalloidin-AlexaFluor488 was included before the final washes, and coverslips were mounted on glass slides with Prolong Gold Antifade reagent (Thermo Fisher Scientific). Cells images were captured using a 63× objective on a Zeiss Axioskop 40 microscope equipped with a Zeiss AxioCam MRm MC100 Spot digital camera and AxioVision software (https://www.micro-shop.zeiss.com/en/us/system/software+axiovision-axiovision+program-axiovision+software/10221/). A minimum of three separate fields were quantified per experiment. Background was established by analysis of cells subjected to the PLA reaction with both HA and Flag antibodies but only transfected with a single vector. When testing for endogenous Sstr2 and Dvl1 interaction, IMR32 cells plated on poly-l-ornithine coated coverslips were washed with DMEM/LH/Hepes and allowed to re-equilibrate at 37 °C, 5% CO_2_ for 15 to 20 min before being treated with 100 nM octreotide for the indicated times or 100 nM PRL2915 for 30 min. Coverslips were washed with cold PBS and fixed with cold methanol for 10 min before performing PLA reactions with mouse anti-Sstr2 and rabbit anti-Dvl1. Control reactions were performed using mouse anti-Sstr2 paired with nonspecific rabbit IgG or rabbit anti-Dvl1 paired with nonspecific mouse IgG. Positive cells were defined as those with a minimum of 6 puncta per cell.

### Cell surface and total receptor ELISA assays

Cell surface receptors were measured using a colorimetric peroxidase assay as described (71). HEK293 cells were plated in poly-l-ornithine (Sigma Aldrich) coated 96-well plates 1 day prior to transfection and used for experiments 1 day after transfection. The day of the experiment, cells were washed with DMEM/LH/Hepes and incubated with primary antibody (mouse anti-HA, 1:10,000, BioLegend) for 2 h at 4 °C. After washing twice with DMEM/LH/Hepes and recovering at 37 °C, 20 min, octreotide (100 nM) was added for the indicated times. Cells were subsequently washed with cold PBS and fixed in 3% PFA in PBS. To visualize the recycled receptor, cells were incubated with goat anti-mouse HRP plus 1% BSA in PBS for 1 h at RT and then incubated with the colorimetric peroxidase substrate, 2,2′-azino-bis(3- ethylbenzthiazoline-6-sulfonic acid) for 45 min. Absorbance was read at 405 nm. The absorbance of untransfected cells was subtracted as background and absorbance was compared to untreated wells on the same plate.

To measure Sstr2 recycling, after cells were incubated with primary antibody (as described for cell surface experiments), they were washed twice and incubated in DMEM/LH/Hepes supplemented with 15 mM NaHCO_3_ (37 °C in a 5% CO_2_ incubator for 20 min). Octreotide (100 nM) was added for 30 min to reach a steady state of internalization (71). Cells were washed twice with assay media and then incubated with fresh media containing 100 nM PRL2915, to inhibit the action of residual remaining agonist. The cells were incubated at 37 °C in 5% CO_2_ for the time points indicated and subsequently fixed with PFA. They were then incubated with secondary antibody, followed by color development with 2,2′-azino-bis(3-ethylbenzthiazoline-6-sulfonic acid) or 3,3′, 5,5;-tetramethylbenzidine.

For determination of endogenous cell surface receptor levels, IMR32 cells were reverse transfected with control and Dvl1 siRNAs and allowed to recover for 36 h before subsequently being trypsinized, counted, and plated at a density of 1 × 10^5^ cells per well coated with poly-l-ornithine. The cells were allowed to attach and grow overnight. The day of the experiment, cells were washed with DMEM/LH/Hepes and incubated with primary antibody (mouse anti-Sstr2, 1:1000 or normal mouse IgG 1 μg/ml) for 2 h at 4 °C. After washing twice with cold DMEM/LH/Hepes and once with PBS, cells were fixed in 3% PFA in PBS. To visualize the surface receptor, cells were incubated with goat anti-mouse HRP plus 1% BSA in PBS for 1 h at RT, washed three times with PBS, then incubated with colorimetric peroxidase substrate for 15 min and stopped with 0.2 M sulfuric acid. Absorbance was read at 450 nm.

### Immunofluorescence microscopy

HEK293 cells were plated on coverslips coated with poly l-ornithine 1 day prior to transfection and then transiently transfected with empty vector, HA-Sstr2, and Flag-Dvl1 as indicated, 1 day prior to experiment. Twenty-four hours post-transfection, the coverslips were washed with DMEM/LH/Hepes and allowed to re-equilibrate at 37 °C, 5% CO_2_ for 15 to 20 min before being treated with 100 nM octreotide for the indicated times. Coverslips were washed with cold PBS and fixed with 3% PFA at RT for 10 min. Cells were permeabilized with 0.3% Triton X-100 in PBS for 15 min at RT. Following 30 min of blocking with 2% BSA in PBS + 0.02% Tween 20 (PBST), coverslips were incubated with primary antibody for 1 h at 37 °C. Coverslips were washed three times with PBST, then incubated with DAPI, secondary antibody, and AlexaFluor-conjugated phalloidin were indicated for 1 h. Coverslips were then washed three times with PBST and once with ddH_2_O before being mounted on glass slides with Prolong Gold Antifade reagent and dried overnight. Cells images were captured using a 63× objective on a Zeiss Axioskop 40 microscope equipped with a Zeiss AxioCam MRm MC100 Spot digital camera and AxioVision software, using a constant exposure time determined by the average of four fields of the brightest coverslips. Random fields were selected for quantification and all cells within each field were quantified. Cell boundaries were identified by the phalloidin signal. For experiments involving Dvl silencing, reverse siRNA transfection was performed 24 h before subsequent transfection of plasmid and cell lysates were analyzed to confirm efficient knockdown.

For imaging endogenous levels of cell surface Sstr2, IMR32 cells were reverse transfected according to the INTERFERin protocol with a final siRNA concentration of 10 nM and plated on poly-l-ornithine coated coverslips. Forty-eight hours after transfection, coverslips were rinsed with serum-free DMEM and incubated with primary antibody (1:250 mouse anti-Sstr2 in DMEM/LH/Hepes) at 4 °C for 2 h. Unbound antibody was removed and the cells were washed and allowed to recover in DMEM/LH/Hepes for 15 min in a CO_2_ incubator before being fixed with 3% PFA. Cells were permeabilized, blocked, and stained with secondary-fluorophore conjugated antibody, DAPI, and phalloidin, as described for HEK293.

For colocalization of HA-Sstr2 and Lamp1 in HEK293 cells, transfection conditions were identical to those above; however, coverslips were fixed with cold methanol rather than 3% PFA. Rabbit anti-LAMP1 was incubated on coverslips overnight at 4 °C, followed by 1 h of incubation with mouse anti-HA at 37 °C. Secondary staining was as usual. Colocalization of HA-Sstr2 and Lysotracker-Red was performed as well. In these experiments, HEK293 cells were reverse transfected with siRNA and allowed to recover overnight; the following day, cells were transfected with pcDNA3.1-HA-Sstr2. On the day of the experiment, cells were treated for 2 h with 50 nM Lysotracker-Red, rinsed twice with PBS, and fixed with 3% PFA in PBS. Subsequent staining was as described above. Remaining cells were harvested for validation of knockdown efficiency. After verification of Dvl silencing by Western blotting, images were acquired with a 60× oil objective on a Nikon A1 confocal microscope controlled by NIS Elements software to assemble z-stacks of a minimum of three fields per condition, with a minimum depth of 4 μm and a slice depth of 0.125 μm.

For analysis of colocalization using Pearson’s coefficient, cell edges were determined by viewing the maximum intensity projection of the entire z-stack. Cells that were positively transected or stained but not saturated in intensity were defined as regions of interest. The Nikon Imaging software-Elements colocalization algorithm was then used for individual z-slices to calculate the Pearson’s coefficient for the regions of interest throughout the volume of the image. A minimum of five cells were quantified per field, with a minimum of three z-stacks per cell. Three replicates of each imaging experiment were performed. Each slice was 0.125 μm and each z-stack was comprised of a minimum of thirty slices. Accordingly, each replicate produced a minimum of 450 data points per condition.

Staining for endogenous Sstr2 and Lamp1 in IMR32 cells was essentially as described above, with mouse anti-Sstr2 (1:200) used as the primary antibody in place of HA. Sstr2 and Lamp1 staining were visualized by confocal microscopy and colocalization was assessed as described above.

### Whole cell cAMP measurements

Whole cell cAMP signaling was analyzed using the cADDis cAMP assay for Gi (Montana Molecular). For measurements with Dvl1 or pan-Dvl silencing, HEK293 cells stably expressing HA-Sstr2 were reverse transfected with control, Dvl1, or pan-Dvl-specific siRNAs and allowed to recover 36 h. Cells were then washed, trypsinized, and counted, then transduced with the Gi-coupled green downward BacMam sensor according to the manufacturer’s instructions, plated in black-wall 96-well plates at 4.8 × 10^4^ cells per well, and allowed to recover overnight. Before measuring cAMP signaling, cells were washed with PBS and allowed to equilibrate at 37 °C. The plate was placed in a Tecan Infinite200 plate reader that was pre-equilibrated at 37 °C. Baseline fluorescence (excitation 483 nm, emission 535 nm) was acquired for 5 min before treating cells with NKH477 (10 μM), NKH477 (10 μM) + octreotide (100 nM), or PBS. Fluorescence was monitored for 15 min after addition of drug. Background cellular fluorescence was measured in no-virus control wells and was subtracted from all values before further analysis. Data were graphed as ΔF (F − F_0_)/F_0_, where F_0_ is the initial fluorescence.

### ERK1/2 activation measurements

The effect of Dvl1 silencing on ERK1/2 signaling was analyzed *via* Western blots. HEK293 cells stably expressing HA-Sstr2 were reverse transfected with control or Dvl1 siRNA and allowed to recover 24 h. Cells were then washed, trypsinized, plated in poly-l-ornithine-coated 6-well dishes, and allowed to recover overnight. The day of the experiment, the cells were washed twice with DMEM/LH/Hepes and equilibrated at 37 °C and 5% CO_2_ for 15 to 20 min, then treated with 100 nM octreotide for the times indicated before harvest with cold PBS, lysis in SDS-urea buffer, and Western blotting.

### GH secretion

For determination of the effect of Dvl1 inhibition on Sstr2-dependent suppression of hormone secretion, GH3 cells were plated at a density of 1 × 10^5^ per well in poly-l-ornithine coated wells of a 12-well dish and allowed to grow 24 h. On the day of the experiment, cells were refed complete medium-containing vehicle or 10 μM Dvl PDZ inhibitor for 4 h. Wells were aspirated and cells were refed medium in the presence or absence of 100 nM octreotide. Cells were incubated for 15 min and subsequently both the supernatant and the cell pellets were harvested. The amount of GH secreted into the medium was determined *via* the Thermo Fisher Scientific rat GH ELISA kit (catalog number KRC5311), following the manufacturer’s instructions. Values were calculated based on the volume of medium harvested per sample and the cell protein content per well. For comparison between experiments, samples were normalized to the vehicle control samples.

### Data and statistical analysis

Data were plotted using Prism 9 (GraphPad) (https://www.graphpad.com/). Statistical significance was determined using unpaired Student’s *t* test, one-way ANOVA, or other appropriate statistics as indicated in the figure legends. A *p* < 0.05 was considered statistically significant.

## Data availability

All data are contained within the article.

## Conflict of interest

The authors declare that they have no conflicts of interest with the contents of this article.

## References

[1] Keskin O., Yalcin S. (2013). A review of the use of somatostatin analogs in oncology. OncoTargets Ther..

[2] Caplin M.E., Pavel M., Ruszniewski P. (2014). Lanreotide in metastatic enteropancreatic neuroendocrine tumors. N. Engl. J. Med..

[3] Pokuri V.K., Fong M.K., Iyer R. (2016). Octreotide and lanreotide in gastroenteropancreatic neuroendocrine tumors. Curr. Oncol. Rep..

[4] Pavel M.E., Sers C. (2016). Women in cancer THEMATIC REVIEW: systemic therapies in neuroendocrine tumors and novel approaches toward personalized medicine. Endocr.Relat. Cancer.

[5] Melmed S., Bronstein M.D., Chanson P., Klibanski A., Casanueva F.F., Wass J.A.H. (2018). A consensus statement on acromegaly therapeutic outcomes. Nat. Rev. Endocrinol..

[6] Hennrich U., Kopka K. (2019). Lutathera((R)): the first FDA- and EMA-approved radiopharmaceutical for peptide receptor radionuclide therapy. Pharmaceuticals (Basel).

[7] Hope T.A., Bodei L., Chan J.A., El-Haddad G., Fidelman N., Kunz P.L. (2020). NANETS/SNMMI consensus statement on patient selection and appropriate Use of (177)Lu-DOTATATE peptide receptor radionuclide therapy. J. Nucl. Med..

[8] Kendi A.T., Halfdanarson T.R., Packard A., Dundar A., Subramaniam R.M. (2019). Therapy with (177)Lu-DOTATATE: clinical implementation and impact on care of patients with neuroendocrine tumors. AJR Am. J. Roentgenol..

[9] Koenig J.A., Edwardson J.M., Humphrey P.P. (1997). Somatostatin receptors in Neuro2A neuroblastoma cells: ligand internalization. Br. J. Pharmacol..

[10] Roosterman D., Roth A., Kreienkamp H.J., Richter D., Meyerhof W. (1997). Distinct agonist-mediated endocytosis of cloned rat somatostatin receptor subtypes expressed in insulinoma cells. J. Neuroendocrinol..

[11] Tulipano G., Stumm R., Pfeiffer M., Kreienkamp H.J., Hollt V., Schulz S. (2004). Differential beta-arrestin trafficking and endosomal sorting of somatostatin receptor subtypes. J. Biol. Chem..

[12] Csaba Z., Lelouvier B., Viollet C., El Ghouzzi V., Toyama K., Videau C. (2007). Activated somatostatin type 2 receptors traffic *in vivo* in central neurons from dendrites to the trans Golgi before recycling. Traffic.

[13] Csaba Z., Dournaud P. (2007). Activated somatostatin type 2 receptors traffic *in vivo* from dendrites to the trans-Golgi network. Ideggyogy. Sz..

[14] Liu Q., Bee M.S., Schonbrunn A. (2009). Site specificity of agonist and second messenger-activated kinases for somatostatin receptor subtype 2A (Sst2A) phosphorylation. Mol. Pharmacol..

[15] Lesche S., Lehmann D., Nagel F., Schmid H.A., Schulz S. (2009). Differential effects of octreotide and pasireotide on somatostatin receptor internalization and trafficking *in vitro*. J. Clin. Endocrinol. Metab..

[16] Poll F., Lehmann D., Illing S., Ginj M., Jacobs S., Lupp A. (2010). Pasireotide and octreotide stimulate distinct patterns of sst2A somatostatin receptor phosphorylation. Mol. Endocrinol..

[17] Ghosh M., Schonbrunn A. (2011). Differential temporal and spatial regulation of somatostatin receptor phosphorylation and dephosphorylation. J. Biol. Chem..

[18] Olsen C., Memarzadeh K., Ulu A., Carr H.S., Bean A.J., Frost J.A. (2019). Regulation of somatostatin receptor 2 trafficking by C-tail motifs and the retromer. Endocrinology.

[19] Romero G., von Zastrow M., Friedman P.A. (2011). Role of PDZ proteins in regulating trafficking, signaling, and function of GPCRs: means, motif, and opportunity. Adv. Pharmacol..

[20] Dunn H.A., Ferguson S.S. (2015). PDZ protein regulation of G protein-coupled receptor trafficking and signaling pathways. Mol. Pharmacol..

[21] Zitzer H., Richter D., Kreienkamp H.J. (1999). Agonist-dependent interaction of the rat somatostatin receptor subtype 2 with cortactin-binding protein 1. J. Biol. Chem..

[22] Kreienkamp H.J., Zitzer H., Richter D. (2000). Identification of proteins interacting with the rat somatostatin receptor subtype 2. J. Physiol. Paris.

[23] Kim J.K., Kwon O., Kim J., Kim E.K., Park H.K., Lee J.E. (2012). PDZ domain-containing 1 (PDZK1) protein regulates phospholipase C-beta3 (PLC-beta3)-specific activation of somatostatin by forming a ternary complex with PLC-beta3 and somatostatin receptors. J. Biol. Chem..

[24] Carr H.S., Chang J.T., Frost J.A. (2021). The PDZ domain protein SYNJ2BP regulates GRK-dependent Sst2A phosphorylation and downstream MAPK signaling. Endocrinology.

[25] Nusse R., Clevers H. (2017). Wnt/beta-catenin signaling, disease, and emerging therapeutic modalities. Cell.

[26] Krishnamurthy N., Kurzrock R. (2018). Targeting the Wnt/beta-catenin pathway in cancer: update on effectors and inhibitors. Cancer Treat. Rev..

[27] Gammons M., Bienz M. (2018). Multiprotein complexes governing Wnt signal transduction. Curr. Opin. Cell Biol..

[28] Tauriello D.V., Jordens I., Kirchner K., Slootstra J.W., Kruitwagen T., Bouwman B.A. (2012). Wnt/beta-catenin signaling requires interaction of the Dishevelled DEP domain and C terminus with a discontinuous motif in Frizzled. Proc. Natl. Acad. Sci. U. S. A..

[29] Koo B.K., Spit M., Jordens I., Low T.Y., Stange D.E., van de Wetering M. (2012). Tumour suppressor RNF43 is a stem-cell E3 ligase that induces endocytosis of Wnt receptors. Nature.

[30] Jiang X., Charlat O., Zamponi R., Yang Y., Cong F. (2015). Dishevelled promotes Wnt receptor degradation through recruitment of ZNRF3/RNF43 E3 ubiquitin ligases. Mol. Cell.

[31] Shan J., Shi D.L., Wang J., Zheng J. (2005). Identification of a specific inhibitor of the dishevelled PDZ domain. Biochemistry.

[32] Arthofer E., Hot B., Petersen J., Strakova K., Jäger S., Grundmann M. (2016). WNT stimulation dissociates a frizzled 4 inactive-state complex with Gα12/13. Mol. Pharmacol..

[33] Lahlou H., Saint-Laurent N., Esteve J.P., Eychene A., Pradayrol L., Pyronnet S. (2003). sst2 Somatostatin receptor inhibits cell proliferation through Ras-, Rap1-, and B-Raf-dependent ERK2 activation. J. Biol. Chem..

[34] Voloshanenko O., Gmach P., Winter J., Kranz D., Boutros M. (2017). Mapping of Wnt-frizzled interactions by multiplex CRISPR targeting of receptor gene families. FASEB J..

[35] Dijksterhuis J.P., Baljinnyam B., Stanger K., Sercan H.O., Ji Y., Andres O. (2015). Systematic mapping of WNT-FZD protein interactions reveals functional selectivity by distinct WNT-FZD pairs. J. Biol. Chem..

[36] Molitch M.E. (2017). Diagnosis and treatment of pituitary adenomas: a review. JAMA.

[37] Badura L., Swanson T., Adamowicz W., Adams J., Cianfrogna J., Fisher K. (2007). An inhibitor of casein kinase I epsilon induces phase delays in circadian rhythms under free-running and entrained conditions. J. Pharmacol. Exp. Ther..

[38] Liu J., Pan S., Hsieh M.H., Ng N., Sun F., Wang T. (2013). Targeting wnt-driven cancer through the inhibition of porcupine by LGK974. Proc. Natl. Acad. Sci. U. S. A..

[39] Peters J.M., McKay R.M., McKay J.P., Graff J.M. (1999). Casein kinase I transduces Wnt signals. Nature.

[40] Sakanaka C., Leong P., Xu L., Harrison S.D., Williams L.T. (1999). Casein kinase iepsilon in the wnt pathway: regulation of beta-catenin function. Proc. Natl. Acad. Sci. U. S. A..

[41] Cong F., Schweizer L., Varmus H. (2004). Casein kinase iepsilon modulates the signaling specificities of dishevelled. Mol. Cell. Biol..

[42] Bernatik O., Ganji R.S., Dijksterhuis J.P., Konik P., Cervenka I., Polonio T. (2011). Sequential activation and inactivation of dishevelled in the Wnt/beta-catenin pathway by casein kinases. J. Biol. Chem..

[43] Davidson G., Wu W., Shen J., Bilic J., Fenger U., Stannek P. (2005). Casein kinase 1 gamma couples Wnt receptor activation to cytoplasmic signal transduction. Nature.

[44] Swiatek W., Kang H., Garcia B.A., Shabanowitz J., Coombs G.S., Hunt D.F. (2006). Negative regulation of LRP6 function by casein kinase I epsilon phosphorylation. J. Biol. Chem..

[45] Harnoš J., Cañizal M.C.A., Jurásek M., Kumar J., Holler C., Schambony A. (2019). Dishevelled-3 conformation dynamics analyzed by FRET-based biosensors reveals a key role of casein kinase 1. Nat. Commun..

[46] Kadowaki T., Wilder E., Klingensmith J., Zachary K., Perrimon N. (1996). The segment polarity gene porcupine encodes a putative multitransmembrane protein involved in wingless processing. Genes Dev..

[47] Reubi J.C., Waser B., Cescato R., Gloor B., Stettler C., Christ E. (2010). Internalized somatostatin receptor subtype 2 in neuroendocrine tumors of octreotide-treated patients. J. Clin. Endocrinol. Metab..

[48] Alshafie W., Pan Y.E., Kreienkamp H.J., Stroh T. (2020). Characterization of agonist-dependent somatostatin receptor subtype 2 trafficking in neuroendocrine cells. Endocrine.

[49] Zebisch M., Xu Y., Krastev C., MacDonald B.T., Chen M., Gilbert R.J. (2013). Structural and molecular basis of ZNRF3/RNF43 transmembrane ubiquitin ligase inhibition by the Wnt agonist R-spondin. Nat. Commun..

[50] Lebensohn A.M., Bazan J.F., Rohatgi R. (2022). Receptor control by membrane-tethered ubiquitin ligases in development and tissue homeostasis. Curr. Top. Dev. Biol..

[51] Soh U.J., Trejo J. (2011). Activated protein C promotes protease-activated receptor-1 cytoprotective signaling through β-arrestin and dishevelled-2 scaffolds. Proc. Natl. Acad. Sci. U. S. A..

[52] Ben-Shlomo A., Melmed S. (2010). Pituitary somatostatin receptor signaling. Trends Endocrinol. Metab..

[53] Theodoropoulou M., Stalla G.K. (2013). Somatostatin receptors: from signaling to clinical practice. Front. Neuroendocrinol..

[54] Elston M.S., Gill A.J., Conaglen J.V., Clarkson A., Shaw J.M., Law A.J. (2008). Wnt pathway inhibitors are strongly down-regulated in pituitary tumors. Endocrinology.

[55] Ren J., Jian F., Jiang H., Sun Y., Pan S., Gu C. (2018). Decreased expression of SFRP2 promotes development of the pituitary corticotroph adenoma by upregulating Wnt signaling. Int. J. Oncol..

[56] Song W., Qian L., Jing G., Jie F., Xiaosong S., Chunhui L. (2018). Aberrant expression of the sFRP and WIF1 genes in invasive non-functioning pituitary adenomas. Mol. Cell. Endocrinol..

[57] Miyakoshi T., Takei M., Kajiya H., Egashira N., Takekoshi S., Teramoto A. (2008). Expression of Wnt4 in human pituitary adenomas regulates activation of the beta-catenin-independent pathway. Endocr. Pathol..

[58] Kim J.T., Li J., Jang E.R., Gulhati P., Rychahou P.G., Napier D.L. (2013). Deregulation of Wnt/β-catenin signaling through genetic or epigenetic alterations in human neuroendocrine tumors. Carcinogenesis.

[59] Bottarelli L., Azzoni C., Pizzi S., D'Adda T., Silini E.M., Bordi C. (2013). Adenomatous polyposis coli gene involvement in ileal enterochromaffin cell neuroendocrine neoplasms. Hum. Pathol..

[60] Estrella J.S., Taggart M.W., Rashid A., Abraham S.C. (2014). Low-grade neuroendocrine tumors arising in intestinal adenomas: evidence for alterations in the adenomatous polyposis coli/β-catenin pathway. Hum. Pathol..

[61] Jiang X., Cao Y., Li F., Su Y., Li Y., Peng Y. (2014). Targeting β-catenin signaling for therapeutic intervention in MEN1-deficient pancreatic neuroendocrine tumours. Nat. Commun..

[62] Uysal-Onganer P., Kawano Y., Caro M., Walker M.M., Diez S., Darrington R.S. (2010). Wnt-11 promotes neuroendocrine-like differentiation, survival and migration of prostate cancer cells. Mol. Cancer.

[63] Yu X., Wang Y., DeGraff D.J., Wills M.L., Matusik R.J. (2011). Wnt/β-catenin activation promotes prostate tumor progression in a mouse model. Oncogene.

[64] Moparthi L., Pizzolato G., Koch S. (2019). Wnt activator FOXB2 drives the neuroendocrine differentiation of prostate cancer. Proc. Natl. Acad. Sci. U. S. A..

[65] Bland T., Wang J., Yin L., Pu T., Li J., Gao J. (2021). WLS-Wnt signaling promotes neuroendocrine prostate cancer. iScience.

[66] Kim M.J., Huang Y., Park J.I. (2020). Targeting wnt signaling for gastrointestinal cancer therapy: present and evolving views. Cancers (Basel).

[67] Resham K., Sharma S.S. (2019). Pharmacological interventions targeting Wnt/β-catenin signaling pathway attenuate paclitaxel-induced peripheral neuropathy. Eur. J. Pharmacol..

[68] Resham K., Sharma S.S. (2019). Pharmacologic inhibition of porcupine, disheveled, and β-catenin in wnt signaling pathway ameliorates diabetic peripheral neuropathy in rats. J. Pain.

[69] Wang C., Dai J., Sun Z., Shi C., Cao H., Chen X. (2015). Targeted inhibition of disheveled PDZ domain via NSC668036 depresses fibrotic process. Exp. Cell Res..

[70] Liu Q., Cescato R., Dewi D.A., Rivier J., Reubi J.C., Schonbrunn A. (2005). Receptor signaling and endocytosis are differentially regulated by somatostatin analogs. Mol. Pharmacol..

[71] Kao Y.J., Ghosh M., Schonbrunn A. (2011). Ligand-dependent mechanisms of sst2A receptor trafficking: role of site-specific phosphorylation and receptor activation in the actions of biased somatostatin agonists. Mol. Endocrinol..

